# Spider venom administration impairs glioblastoma growth and modulates immune response in a non-clinical model

**DOI:** 10.1038/s41598-020-62620-9

**Published:** 2020-04-03

**Authors:** Amanda Pires Bonfanti, Natália Barreto, Jaqueline Munhoz, Marcus Caballero, Gabriel Cordeiro, Thomaz Rocha-e-Silva, Rafael Sutti, Fernanda Moura, Sérgio Brunetto, Celso Dario Ramos, Rodolfo Thomé, Liana Verinaud, Catarina Rapôso

**Affiliations:** 1 Faculdade de Ciências Farmacêuticas, Universidade Estadual de Campinas (UNICAMP), Campinas, SP Brazil; 2 Departamento de Biologia Estrutural e Funcional, Instituto de Biologia, UNICAMP, Campinas, SP Brazil; 3 Faculdade Israelita de Ciências da Saúde Albert Einstein, São Paulo, SP Brazil; 4 Faculdade de Ciências Médicas, Santa Casa de São Paulo, São Paulo, SP Brazil; 5 Faculdade de Ciências Médicas, UNICAMP, Campinas, SP Brazil; 6 Centro de Engenharia Biomédica, UNICAMP, Campinas, SP Brazil; 7 Serviço de Medicina Nuclear, Hospital das Clínicas, UNICAMP, Campinas, SP Brazil; 8 Department of Neurology, Thomas Jefferson University, Philadelphia, PA USA

**Keywords:** Cancer immunotherapy, Pharmacology

## Abstract

Molecules from animal venoms are promising candidates for the development of new drugs. Previous *in vitro* studies have shown that the venom of the spider *Phoneutria nigriventer* (PnV) is a potential source of antineoplastic components with activity in glioblastoma (GB) cell lines. In the present work, the effects of PnV on tumor development were established *in vivo* using a xenogeneic model. Human GB (NG97, the most responsive line in the previous study) cells were inoculated (s.c.) on the back of RAG^−/−^ mice. PnV (100 µg/Kg) was administrated every 48 h (i.p.) for 14 days and several endpoints were evaluated: tumor growth and metabolism (by microPET/CT, using ^18^F-FDG), tumor weight and volume, histopathology, blood analysis, percentage and profile of macrophages, neutrophils and NK cells isolated from the spleen (by flow cytometry) and the presence of macrophages (Iba-1 positive) within/surrounding the tumor. The effect of venom was also evaluated on macrophages *in vitro*. Tumors from PnV-treated animals were smaller and did not uptake detectable amounts of ^18^F-FDG, compared to control (untreated). PnV-tumor was necrotic, lacking the histopathological characteristics typical of GB. Since in classic chemotherapies it is observed a decrease in immune response, methotrexate (MTX) was used only to compare the PnV effects on innate immune cells with a highly immunosuppressive antineoplastic drug. The venom increased monocytes, neutrophils and NK cells, and this effect was the opposite of that observed in the animals treated with MTX. PnV increased the number of macrophages in the tumor, while did not increase in the spleen, suggesting that PnV-activated macrophages were led preferentially to the tumor. Macrophages were activated *in vitro* by the venom, becoming more phagocytic; these results confirm that this cell is a target of PnV components. Spleen and *in vitro* PnV-activated macrophages were different of M1, since they did not produce pro- and anti-inflammatory cytokines. Studies in progress are selecting the venom molecules with antitumor and immunomodulatory effects and trying to better understand their mechanisms. The identification, optimization and synthesis of antineoplastic drugs from PnV molecules may lead to a new multitarget chemotherapy. Glioblastoma is associated with high morbidity and mortality; therefore, research to develop new treatments has great social relevance. Natural products and their derivatives represent over one-third of all new molecular entities approved by FDA. However, arthropod venoms are underexploited, although they are a rich source of new molecules. A recent *in vitro* screening of the *Phoneutria nigriventer* spider venom (PnV) antitumor effects by our group has shown that the venom significantly affected glioblastoma cell lines. Therefore, it would be relevant to establish the effects of PnV on tumor development *in vivo*, considering the complex neoplastic microenvironment. The venom was effective at impairing tumor development in murine xenogeneic model, activating the innate immune response and increasing tumor infiltrating macrophages. In addition, PnV activated macrophages *in vitro* for a different profile of M1. These activated PnV-macrophages have potential to fight the tumor without promoting tumorigenesis. Studies in progress are selecting the venom molecules with antitumor and immunomodulatory effects and trying to better understand their mechanisms. We aim to synthesize and carry out a formulation with these antineoplastic molecules for clinical trials. Spider venom biomolecules induced smaller and necrotic xenogeneic GB; spider venom activated the innate immune system; venom increased blood monocytes and the migration of macrophages to the tumor; activated PnV-macrophages have a profile different of M1 and have a potential to fight the tumor without promote tumorigenesis.

## Introduction

Glioblastoma (GB) is the most common and aggressive primary malignant brain tumor in adults, accounting for more than half of malignant gliomas^1^. Although rare, they are associated with high morbidity and mortality, and few patients achieve long-term survival of 2.5 years. The relative survival for the first year is 35%, 13.7% for the second year and less than 5% surviving for 5 years after diagnosis^2,3^. A multimodal approach including maximal surgical resection, radiotherapy and adjuvant chemotherapy with temozolomide or carmustine is the standard therapy (for patients with newly diagnosed GB and younger than 70 years old)^4^. However, recurrence seems to be the rule, despite the standard care. Notwithstanding efforts to find better treatment, it remains an incurable disease with a poor survival rate.

Natural products have greatly contributed to the history of drugs development and identification of new molecular entities. Patridge *et al*.^5^ assessed all FDA-approved new molecules, revealing that natural products and their derivatives represented over one-third of those new entities. Nearly one-half of these are derived from mammals, one-quarter from microbes and one-quarter from plants. On the other hand, the venoms of arthropods (mainly spiders and scorpions) are an underexploited source of new molecular entities. These venoms are a mixture of biologically active molecules, such as proteins and non-protein substances, with specific targets in cells and tissues^6^, representing promising compounds to guide the development of drugs. Hence, biomolecules in scorpion and spider venoms have been shown to affect the cancer hallmarks (see Rapôso^7^ for a comprehensive review).

The venom of the spider *Phoneutria nigriventer* (PnV) (Ctenidae, Araneomorphae), from South America, contains potent basic peptides, some of them neurotoxic^8^. PnV has been shown to permeate the blood–brain barrier (BBB)^9–12^ and affect astrocytes, inducing profound alterations in their morphology and cytoskeleton^13^. Such data opened room for the hypothesis that the venom could have molecular targets in glioma cells.

In fact, a recent *in vitro* screening of the PnV antitumor effects has shown that the venom is a potential source of cancer drug candidates^14^. The venom significantly affected all of the tumor cell lines studied, although the non-glioma tumor cell (HeLa – cervical tumor line) was less sensitive than GB cell lines (NG97 and U-251). A clinically relevant point is that the venom had no cytotoxic effect on non-tumor cells (fibroblasts L929 line). In the present study, the effects of PnV on tumor development were established *in vivo*, in addition to analyzing the innate immune response and the prognosis, using a xenogeneic murine model.

## Materials and Methods

### Reagents and venom

All the chemicals were obtained from Sigma Aldrich (St. Louis, MO), unless otherwise stated. Two samples of crude lyophilized venom of *P. nigriventer* were obtained by electrical stimulation of numerous adult spiders (male and female). The quality and reproducibility of the venom were evaluated by high pressure liquid chromatography (HPLC) in a reverse phase C18 column monitored at 214 nm. The lyophilized venom was stored at −80 °C and dissolved immediately prior to use.

### Animal care, tumor implant and treatments

All experiments were conducted in accordance with the Ethical Principles on Animal Research, adopted by the Brazilian College on Animal Experimentation (Colégio Brasileiro de Experimentação Animal – COBEA), with the prior approval of the Ethics Committee on the Use of Animals (CEUA) of the Universidade Estadual de Campinas (UNICAMP) (#4603-1/2017). The use of Genetically Modified Organism (GMO) was authorized by the Internal Biosafety Committee of UNICAMP (CIBio) (#2017/02). The animals were kept in the Animal Facility of the Biology Institute, Department of Functional and Structural Biology, UNICAMP.

Immunodeficient C57BL6 RAG^−/−^ (B6.129S7-Rag1tm1Mom) mice, females, 8 to 12 weeks old, were used to perform the dorsal subcutaneous xenogeneic tumor model. Human glioblastoma NG97 cells were donated by a patient from the Hospital of the UNICAMP and the cell line was established and characterized in a sequence of published studies^15–19^. 3 × 10^5^ cells were suspended in 100 μL of PBS and injected subcutaneously into the dorsal flank of mice. After 7 days of cells inoculation, the animals were randomly divided into three groups (n = 6–8 per group): control (treated with 100 μL sterile 0.9% saline – vehicle – intraperitoneal every 48 hours), PnV (treated with venom, 100 µg/Kg in 100 µL, intraperitoneally every 48 hours) and MTX (treated with methotrexate, 5 mg/Kg in 100 µL, intramuscularly every 48 hours; this group was made only to compare the blood parameters). The treatments were performed for 14 days; therefore, the total duration of the experiment was 21 days. Throughout the experiment, the animals were monitored daily for body weight and survival. At the end, mice were euthanized and the tumors were excised for weight and volume recording and other analysis described below.

### Micro PET-CT (Positron Emission Tomography and Computed Tomography)

The micro-PET/CT scan (AlbiraSi, Bruker) was performed on the day 21 (at the end of the treatments), at the Preclinical Image Laboratory (LIP) - Medical Sciences School, UNICAMP. The equipment has three attached image modes: PET, SPECT and CT. For this research, the PET and CT modalities were used. Prior to administration of the radiotracer ^18^F-FDG (fluorodeoxyglucose), mice were fasted for at least 6.0 h. The ^18^F-FDG was injected into the intraorbital plexus (i.v.) 40 min before the scan start, for the biodistribution of the radiotracer. The animal was placed in a dedicated isoflurane administration system (MIP/Anesthesia Technologies) for inhalation anesthesia. With no further motor stimuli, the radiotracer was administered and throughout the experiment the animal was maintained under isoflurane and oxygen administration. In addition, the temperature of the animal’s environment was constantly checked, reaching about 23 °C (73.4 °F). The protocols chosen (selected) to acquire the images of the animals were: PET in single mode of 20 minutes and CT of 35 kV, 400 uA and 1024 projections. The images were reconstructed in the AlbiraSi system reconstruction mode. For CT, two images were generated: CT Low (for attenuation correction) and CT High (for corregistration and fusion). For PET, the MLEM (Maximum Likelihood Estimation Method), with voxel size 20 × 20 × 0.5, 12 iterations and with all the corrections activated (scatter correction, decay correction and attenuation correction with CT Low), was used. Finally, for the quantification of the images, the PET fusion image with attenuation correction and CT High were used. To quantify the uptake of the radiotracer present in the volume, the SUV calculation (standardized uptake value) is adopted. This metric can be defined as the ratio between the concentration of activity present in the structure (Bq/volume) and the activity injected (Bq) in the individual multiplied by its respective mass (grams for animals and kilograms for humans). In the present work, the SUV average was quantified, that is, the value of the average concentration present in the structure of interest.

### Tumor histopathology

After PET-CT, the animals were anesthetized with ketamine (80 mg/Kg) and xylazine (10 mg/Kg) and sacrificed. The tumors were dissected and fixed in phosphate-buffered 10% formalin. Samples were then dehydrated by graded ethanol and embedded in Paraplast Plus. Sections of 5 µm were examined after staining with hematoxylin and eosin (H&E).

### Blood analysis – blood cells and toxicity-related parameters

After euthanasia, blood was collected for hemogram and serum was used for determination of markers of hepatic dysfunction (glutamic-oxaloacetic transaminase – GOT; and glutamic pyruvic transaminase – GPT) and renal dysfunction (creatinine and urea). Blood tests were performed by a specialized veterinary laboratory (Echoa Laboratory, Campinas, SP, Brazil).

### Flow cytometry of circulating immune cells

The spleen was collected from all mice to evaluate macrophages, neutrophils and natural Killer (NK) surface proteins and cytokines expression. Spleens were individually homogenized in 1 mL of sterile PBS (0.02 M phosphate buffered saline) using 70 µm cell strainer. After viability testing with Trypan Blue, the cells were incubated with PMA (Phorbol 12-myristate 13-acetate, 1 mg/mL), Ionomycin (5 mg/mL) and Brefeldin A (5 mg/mL) for 4 h, at 37 °C, and the immunostaining was done with the following antibodies (all from eBioscience, USA): anti-CD11b (Clone M1/70, PE, #557397) and anti-F4/80 (Clone BM8, PE-Cy7, #25-4801-82), incubated for 30 minutes (4 °C, in a dark chamber) (anti-Ly6G/Ly6C Clone RB6-8C5, PE, #553128, and anti-NK1.1 Clone PK136, PE, #553165 were also done and are available in the Supplemental Material). The cells were then fixed with 1% paraformaldehyde and permeabilized with specific buffer. After, cells were incubated with antibodies against the cytokines: IFN-γ (Clone XMG1.2, APC, #126406) and IL-10 (Clone JES5-16E3, PerCP Cy5.5, #505028) (overnight, at 4 °C, in dark chamber). Cells were acquired using flow cytometer and data were analyzed by FlowJo VX software.

### Immunofluorescence: detection of tumor associated macrophages

The level of Iba-1 (a macrophage marker) was investigated in 5 μm paraffin sections. Sections were deparaffinized and boiled in citrate buffer (1 mM; pH 6.0) for 30 min in a steam cooker for antigenic recovery. Rabbit polyclonal antibody against Iba-1 (1:100 dilution, FUJIFILM Wako Chemicals U.S.A Corp, Richmond, VA, EUA, #019-19741) was used as primary antibody (negative controls were maintained in PBS), followed by secondary anti-rabbit Cy5 conjugated (Jackson Immuno Research, #711-175-152). Images were acquired using a microscope (Leica DM5500B) coupled to a camera (DFC345 FX) and Image-ProPlus 6.0 software (Media Cybemetics, USA).

### Cell culture and treatments

Macrophages were obtained from differentiation of murine bone marrow cells cultivated with L929 cells supernatant, according to Marim *et al*.^20^. Bone marrow precursors were collected from femurs of 7–10 weeks old male C57BL/6 J mice. After collection, the marrow cell suspension was cultured in Petri dishes with 10 mL of Iscove’s Modified Dulbecco’s Media (IMDM) supplemented with 20% fetal bovine serum (FBS) and 30% L929 cell supernatant (as a source of M-CSF). After four days, another 10 mL of fresh medium was added in the same proportions. This culture was incubated for seven days at 37 °C and 5% of CO_2_ and thereafter these cells were washed with PBS and adherent cells (differentiated macrophages) were harvested with a cell scraper and transferred to a 24 wells plate (2 × 10^5^ cells per well) with a round cover slip at the bottom.

### Phagocytosis assay

For this test, the pHrodo Red E. coli BioParticles Conjugate for Phagocytosis assay was used (Thermo Fisher Scientific, #P35361). The macrophages were pre-activated with 20 ng/mL recombinant mouse IFN-γ (BD #554587) for 48 h and then received the following treatments: PBS only (M0 profile control), E. coli O26:B6 LPS 1 µg/mL (used as a canonical positive control of macrophages activation to M1 profile^21^ to compare with PnV effects), or PnV 14 µg/mL. After 24 h, treatments were removed, macrophages were washed with PBS and incubated for 4 h with 50 µg bioparticles. The cells were then extensively washed, fixed with paraformaldehyde for 15 min and labeled with Iba-1 by immunofluorescence. Briefly, cells were incubated with blocking buffer (PBS with 1% BSA and 0.3% Triton X100) for 2 h, followed by anti-Iba-1 (Wako #019-19741; 1:700) overnight, and secondary antibody (Cy2-Goat anti-rabbit, Jackson ImmunoResearch, #111225144; 1:1000) for 1 h. Nuclei were stained with DAPI (1:1000) for 5 min. Macrophages were washed and sliders were mounted using a mounting medium for fluorescence. Images were acquired in the Cytation 5 – Cell Imaging Multi-Mode reader (BioTek, Winooski, VT, USA). For quantification, images of three randomly selected fields per treatment were taken and integrate analysis of pixels was determined using Image J software (version 1.52p, NIH, USA).

### ELISA (enzyme-linked immunosorbent assay)

The quantification of TNF-alpha and IL-10 in the culture medium was performed using R&D Systems – DuoSet immunoassay kits in accordance with the manufacturer’s instructions. Monoclonal antibodies specific for mouse TNF-alpha and IL-10 were pre-coated on the microplate. Standards, controls and samples were pipetted into the wells and any TNF-alpha or IL-10 present was bound to the immobilized antibody. Following washing, a substrate solution was added. The enzyme reaction yields a blue product that turns yellow when the stop solution is added. The intensity of the color measured is in proportion to the amount of mouse cytokines bound at the initial steps. The sample values were then read off the standard curve in a Multiskan Go microplate spectrophotometer (Thermo Fisher Scientific, Inc., Waltham, MA, USA). All dosages were performed in triplicate.

### Venom purification and MTT (cell viability assay)

The initial fractionation of the crude venom was performed by the Amicon Ultra Centrifugal Filter (#UFC801008; Thermo Fisher Scientific, Suwannee, GA). This procedure consisted of separating crude venom by molecular mass using molecular filters, generating three main fractions named: F1 (low weight, less than 3 kDa), F2 (intermediate weight, between 3 and 10 kDa) and F3 (high weight, more than 10 kDa). From these, cell viability assay (MTT; described below) was conducted to select the most significant fraction, considering the antineoplastic effects; Then, F1 and F2 were chosen and F3 was eliminated. Further purification of F1 and F2 was carried out: reversed phase HPLC was performed using a Shimadzu VP-ODS column, 0.1% trifluoroacetic acid (TFA) as mobile phase and 90% acetonitrile 0.1% TFA as eluent. More purified components were obtained, named subfractions 1–12 (SF1-SF12).

Thiazolyl Blue Tetrazolium Bromide (MTT) was used to determine if Fs and SFs would alter cell viability. NG97 cells were seeded in 96-well plates at an initial density of 5 × 10^5^ cells per well and incubated for 72 h at 37 °C for confluence (in IMDM supplemented with 10% FBS and 100 UI/ml penicillin and streptomycin). Then, cells were treated with PnV (14 µg/ml), F1, F2, F3 or all twelve subfractions (SF1-SF12; 0.1 µg/ml) for 5 h, while control cells were maintained in medium. After removal of the treatments, MTT was added to each well and incubated at 37 °C for 4 h according to the manufacturer’s protocol. Thereafter, acidified isopropanol was added to each well to solubilize the blue formazan crystals. Absorbance at 540 nm was determined on a Multiskan GO spectrophotometer.

### Statistic

Values were analyzed using the GraphPad Prism software package (San Diego, CA, USA; v. 5.0). The level of significance was obtained by one-way analysis of variance (ANOVA) followed by Dunnett’s multiple comparisons test. Error bars show the standard error of the mean (SEM). Unpaired Student’s t-test was used to compare each treatment with the control. A p-value <0.05 indicated statistical significance.

## Results

### Quality and reproducibility of venom

HPLC demonstrated that there was no significant difference between the two extracted venom samples. The samples were the same used in the experiments published before by Santos *et al*. [14], however the quality of PnV lots was established using 214 nm wavelength (Supplementary Fig. 1).

### PnV administration increased body weight gain and improved survival, compared to control

**Figure 1:**
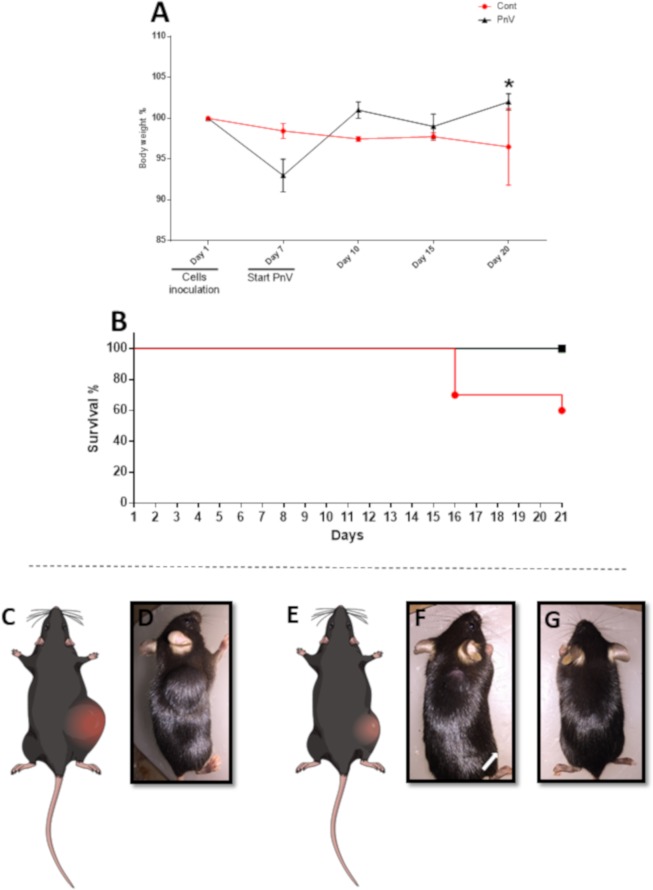
Body weight variation and survival. (**A**) – The graph shows the average body weight gain in untreated (control) and PnV-treated mice (red and black lines, respectively). The first weight (day 1) was considered zero (no gain). After 7 days of cells inoculation, treatments were initiated (day 7). At day 21, PnV treated animals significantly recovered the lost body weight, compared to day 7 (*P < 0.05). (**B**) – Graph showing the survive during the experiment. The control group presented 40% mortality, whereas PnV treated group did not show death (100% survival). (**C–G**) Schematic illustrations and photos of representatives RAG^−/−^ mice with tumor implanted into the back. (**C**,**D**) - Control animal (untreated) 21 days after inoculation of GB (NG97) cells, showing a large tumor. (**E**,**F**) - PnV-treated animal 21 days after inoculation of GB (NG97) cells, showing a smaller tumor; some PnV-treated animals did not develop tumor mass (**G**). Arrows in B and D indicate the tumor.

The control group recorded gradual loss of weight (data were not significant) after inoculation of the cells (day 1) until the end of the experiment (day 20, last recorded weight). On day 7, the treatments were started. The PnV group presented weight loss on day 7 in relation to day 1, followed by progressive recovery after the beginning of the treatment, being significant when comparing day 20 with day 7 (p < 0,05) (Fig. 1A). Control animals showed 40% of mortality (due to general clinical condition caused by the tumor), while PnV group had no deaths (Fig. 1B).

### Mice treated with PnV developed smaller and ^18^F-FDG-negative tumors, compared to control and MTX

**Figure 2:**
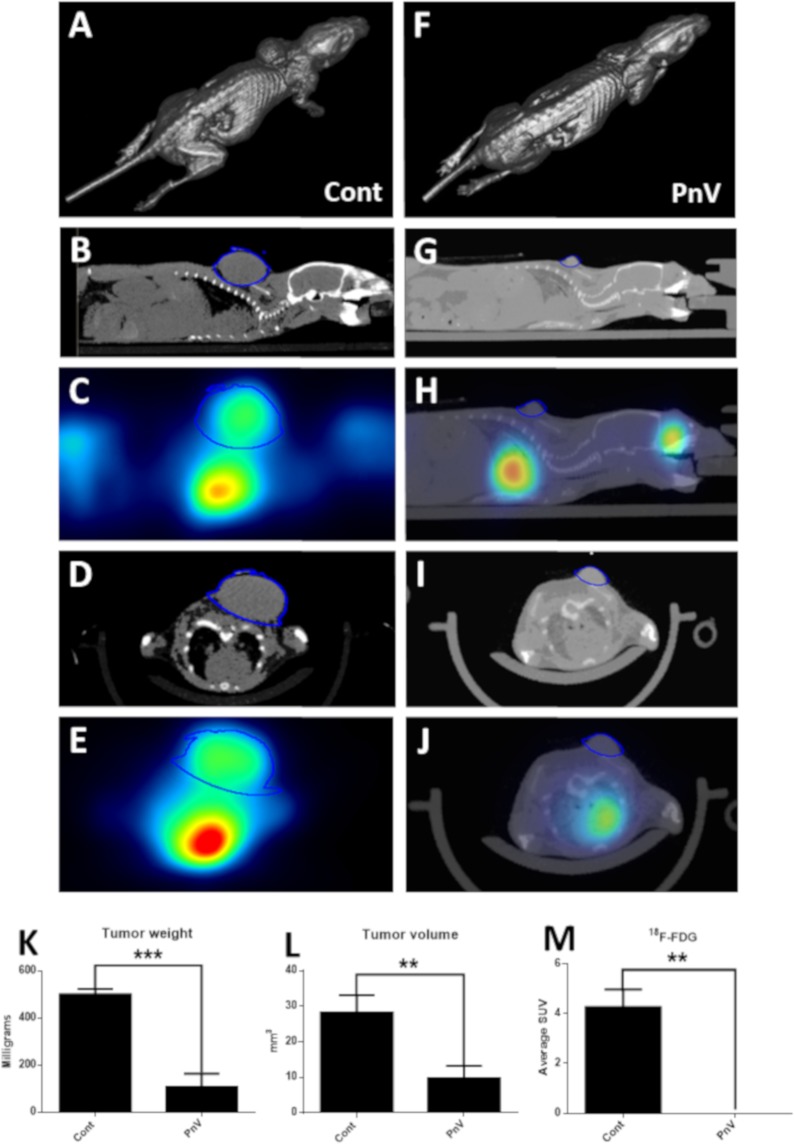
Positron Emission Tomography-Computed Tomography (PET-CT) 21 days after inoculation of GB (NG97) cells. (**A–E**) Control animal (untreated). (**F–J**) PnV-treated animal. A and F are reconstructed 3D images; (**B–H**) are sagittal sections; (**D–J**) are transversal sections. The tumor boundaries are surrounded by a line. Only the PnV-treated animal was demonstrated by the CT and PET overlap, since there was no uptake of ^18^F-FDG (panels H and J). Graphs K and L show, respectively, statistical analysis of tumor weight and volume (measured after dissecting). Graph G shows ^18^F-FDG uptake (SUV - Standardized Uptake Value). **p < 0.01, ***p < 0.001.

Figure 1(C–G) shows representative animals (schematically and per photograph) of the control (C and D) and PnV (E - G), at the end of the experiment (day 21). PET-CT images showed that control animals developed large tumors (Fig. 2A,B,D), with intense uptake of ^18^F-FDG (Fig. 2C,E), suggesting a high metabolism, expected for these tumor cells. Mice receiving PnV therapy showed smaller tumors (Fig. 2F,G,L), which were negative for ^18^F-FDG (Fig. 2H,J), indicating slower metabolism compared to control. Figure 2A,B represent 3D reconstructions of CT for each group. The graphs K and L of Fig. 2 show the statistic results of tumor weight and volume, respectively, and graph M shows the results of ^18^F-FDG uptake (SUV average). Representative animals of control and PnV groups are also shown by 3D animation. Table 1 shows the weight and volume of individual tumors.

**Table 1:** Weight and volume of tumors.

Animal	Tumor weight (mg)	Tumor volume (mm^3^)
Control 1	582.7	15.8
Control 2	—	—
Control 3	434.1	22.1
Control 4	501.7	15.8
Control 5	556.1	35.8
Control 6	—	—
Control 7	459.2	35.7
Control 8	—	—
Control 9	472.0	44.2
Control 10	—	—
PnV 1	0.0	0.0
PnV 2	0.0	0.0
PnV 3	34.0	6.3
PnV 4	29.5	7.4
PnV 5	580.0	33.7
PnV 6	32.7	6.3
PnV 7	122.4	9.5
PnV 8	113.0	12.6
PnV 9	0.0	0.0
PnV 10	178.2	22.1

Four control animals died during the experiment and tumor data were not considered.

Supplementary Figure 2 shows a PnV-treated animal that did not develop tumor in the back (the site of cells inoculation), however, interestingly, only this mouse developed a brain tumor, as shown by CT (B - D) and PET-^18^F-FDG (E - G) (panel A represents a 3D reconstruction).

### Chemotherapy with PnV induced tumor necrosis

**Figure 3:**
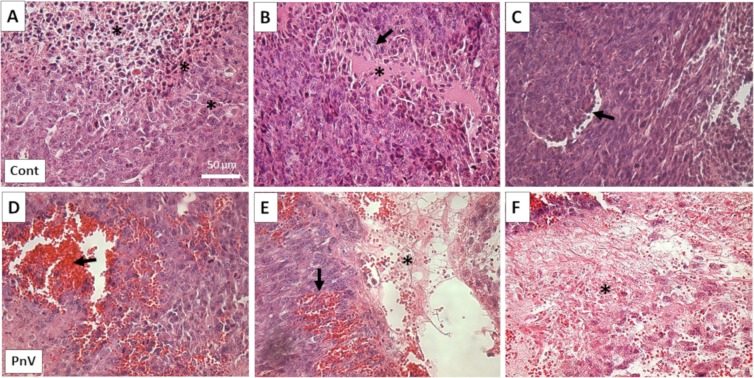
Histopathology – Representative tumor sections stained with hematoxylin and eosin. (**A–C**): Control animal; (**D–F**): PnV-treated animal. Arrows and asterisks show typical characteristics of glioblastoma (see the main text for details). Note the extensive necrosis on the PnV tumor (asterisks in panels E and F). All figures have the same scale (bar shown in panel A only).

Histopathological analysis has showed that control animals developed tumors with typical GB characteristics (Fig. 3A–C). Panel A of Fig. 3 shows a hypercellular tumor composed of a variety of cell types (cell polymorphism) (asterisks), including small cells with hyperchromatic nuclei, cells with pink glial-like cytoplasm, and endothelial cells. In panel B, it is possible to see an area with a pink cellular necrosis at the center (asterisk) with tumor cell palisading (arrow); also, glomeruloid vascular proliferation of endothelial cells is present (arrow on panel C).

Tumors of animals treated with PnV have lost most of the typical characteristics of glioblastoma; It has showed many congestive blood vessels (arrow in panels D and E of Fig. 3) and large areas of necrosis (asterisks in panels E and F).

### Treatment with PnV altered blood cell count, leading to a different profile compared to MTX

**Figure 4:**
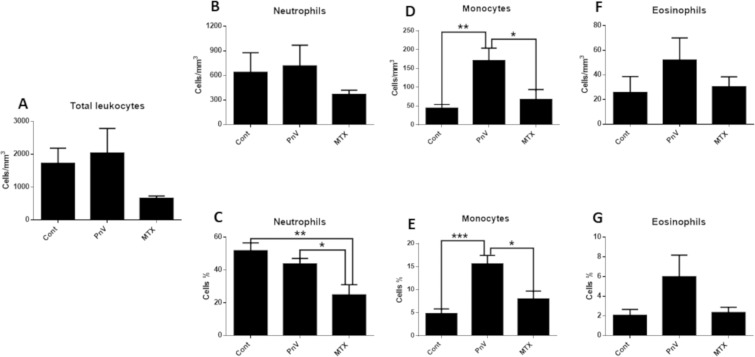
White blood cell count. (**A**) - Treatments did not significantly alter the number of total leucocytes. (**B**,**C**) - The number of neutrophils per mm^3^ was not significantly changed (**B**), however MTX significantly decreased the cell percentage (**C**). (**D**,**E**) - PnV significantly increased the number of monocytes per mm^3^ (**D**) and percentage (**E**). The treatments did not altered eosinophils counts (**F**,**G**). *p < 0.05, **p < 0.01, ***p < 0.001.

In general, chemotherapy with PnV induced an increase in blood cells, whereas MTX induced a decrease (expected for traditional chemotherapy) (Fig. 4); The treatments did not significantly alter the total leucocytes, however it can be observed that, qualitatively, PnV-treated animals were prone to increase them, while MTX showed a decrease (Fig. 4A). The same profile was observed for the number of neutrophils (Fig. 4B). The percentage of cells, on the other hand, showed that PnV did not induce change, whereas MTX induced a significant neutropenia (Fig. 4C). Treatment with PnV induced a significant increase in the number of monocytes and percentage of cells (compared to control and MTX) (Fig. 4D,E). For eosinophils, no significant alterations were observed (Fig. 4F,G).

**Figure 5:**
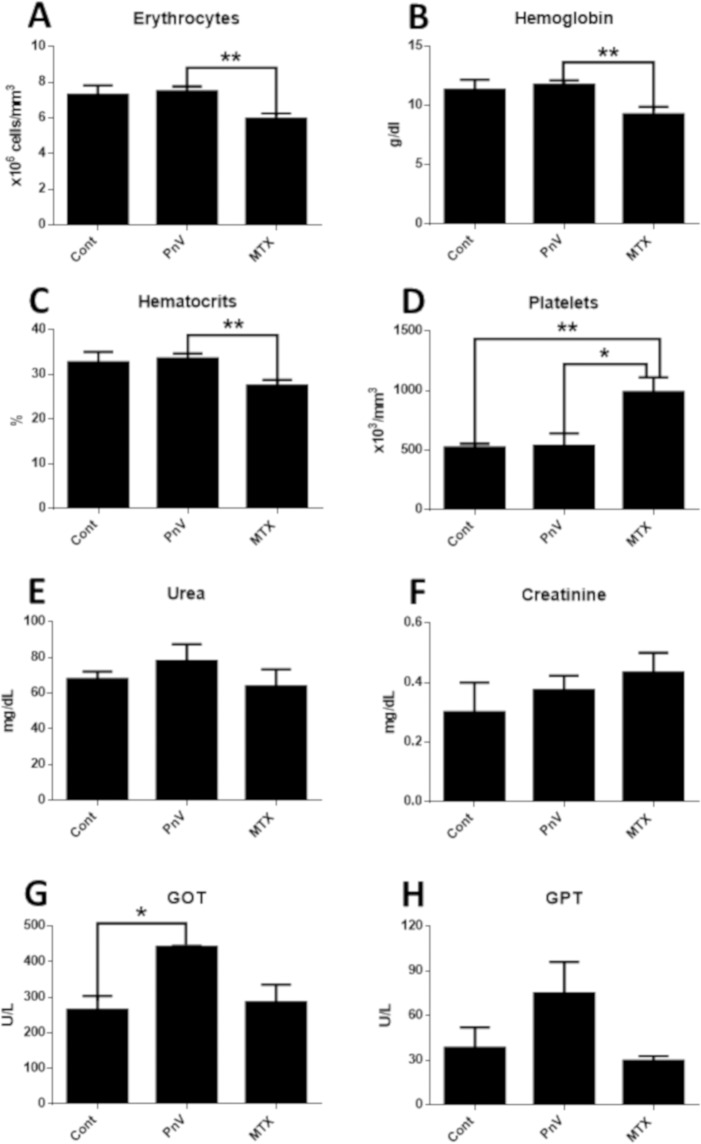
(**A–D**) Red blood cell and platelet count. Red blood cells (**A**) hemoglobin (**B**) and hematocrits (**C**) were significantly decreased in animals receiving MTX, compared to mice treated with PnV. The administration of MTX significantly increased platelets compared with all other groups (**D**). (**E–H**) Serum dosage of renal (urea and creatinine) and hepatic (GOT and GPT) functional markers. (**E**,**F**) – There were no significant changes in urea and creatinine dosages. (**G**,**H**) – PnV significantly increased GOT, comparing to control, but GPT was not altered by the treatments. *p < 0.05, **p < 0.01.

Compared to the control, PnV did not change the number of erythrocytes. However, the number of red cells was significantly lower in the animals receiving MTX (Fig. 5A). A similar result was observed for hemoglobin and hematocrit (Fig. 5B,C). PnV did not alter the platelets, comparing to the control animals. MTX, on the other hand, induced a significant increase in platelets compared to all other groups (Fig. 5D).

### Chemotherapy with PnV induced alterations in hepatic, but not in kidney, functional parameters

Hepatic function markers, GOT and GTP, were also measured in serum (Fig. 5E,F). PnV induced a significant increase of GOT, compared to control, whereas MTX did not lead to alterations of this enzyme. The level of GTP was not significantly altered by the drugs compared to the control, although the venom tended to increase this marker. Markers of renal function, urea and creatinine, were measured (Fig. 5G,H, respectively); the treatments did not induce alteration in the serum levels of these markers.

### PnV did not change total macrophages from spleen, but decreased macrophages expressing IFN-γ and IL-10. The venom increased spleen neutrophils and natural killer (NK) cells

**Figure 6:**
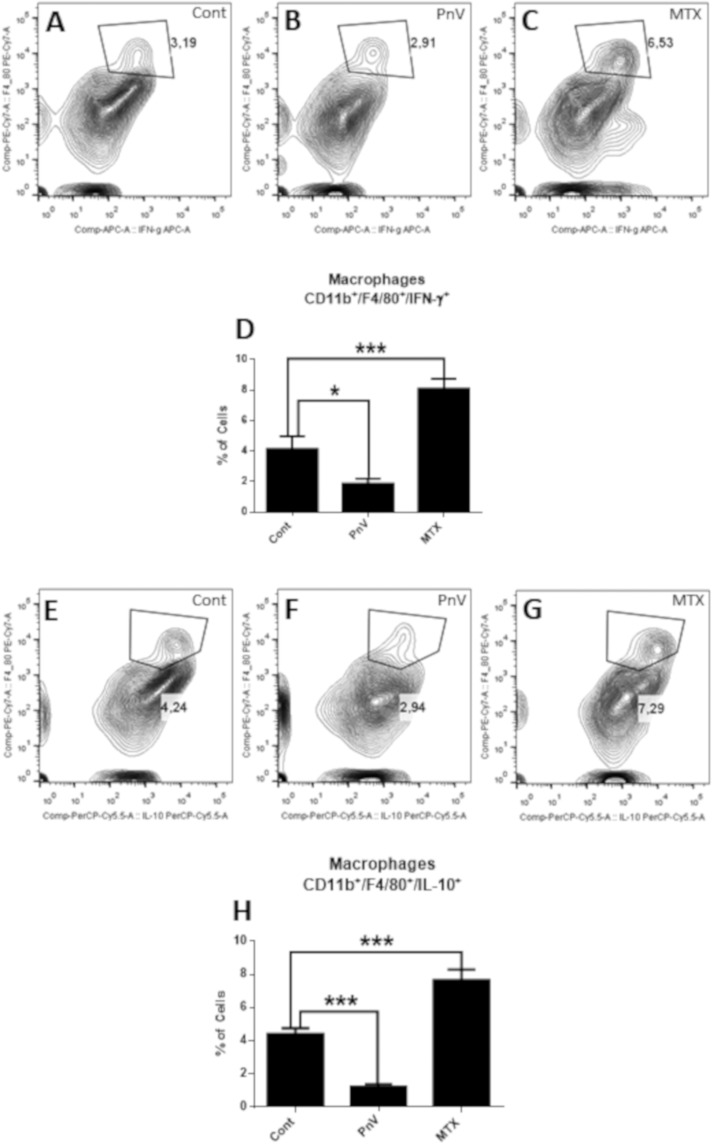
Flow cytometry of activated (F4/80^+^ and IFN-γ^+^) and suppressor (F4/80^+^ and IL-10^+^) macrophages isolated from the spleen. (**A–C**) Representative histograms of activated macrophages. (**E–G**) Representative histograms of suppressor macrophages. (**D–H**) – Graphs showing statistical analysis. PnV administration decreased the percentage of both macrophage profiles compared to control, while MTX increased them. *p < 0.05, ***p < 0.001.

The percentage of total macrophages (CD11b and F4/80 positive cells) isolated from the spleen were not altered by the treatment with venom, in comparison to the control group (data not shown). On the other hand, the percentage of macrophages (CD11b and F4/80 positive) expressing IFN-γ (activated/proinflammatory) (Fig. 6A–D) was strongly decreased by PnV therapy, compared to control. Interestingly, a similar result was observed for IL-10 expressing macrophages (suppressor/anti-inflammatory) (Fig. 6E–H). On the other hand, MTX had the opposite effect, significantly increasing macrophages in both profiles.

**Figure 7:**
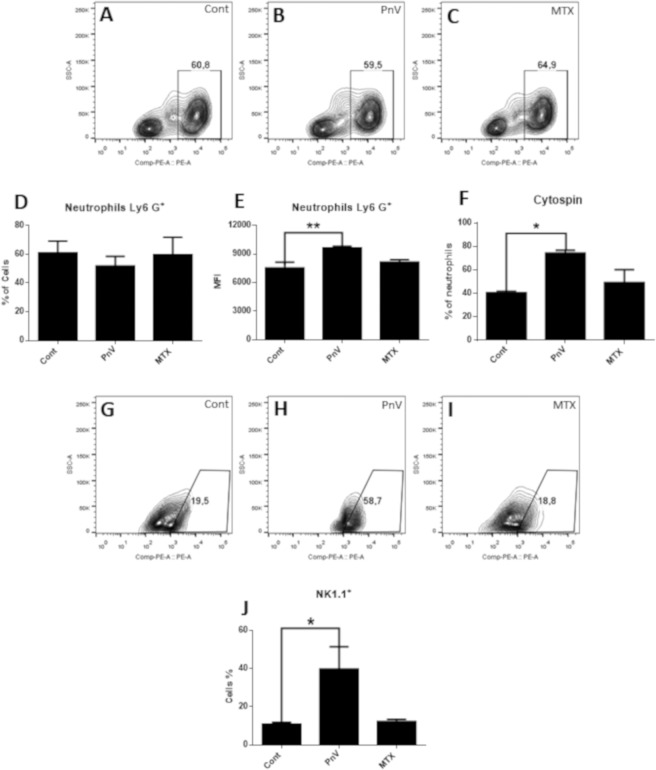
Flow cytometry of neutrophils (Ly6^+^) and Natural Killer cells (NK1.1^+^). (**A–C**): Representative histograms of neutrophils. (**D**,**E**) – Graphs showing statistical results; PnV treatment did not change the percentage of cells (**D**), however increased the MFI (mean fluorescence intensity) (**E**), compared to control. (**F**) – Graph showing the results of cytospin; PnV induced a significant increase in the percentage of neutrophils, compared to the control. (**G–I**) Representative histograms of NK cells. (**J**) – Graph showing statistical results; PnV significantly increased the percentage of NK cells, compared to control. MTX did not change neutrophils and NK cells in comparison to control cells. *p < 0.05, **p < 0.01.

Figure 7 shows that the treatment with PnV did not alter the percentage of neutrophils, compared to control (histograms A–C, and graph D); however, the MFI (Mean Fluorescence Intensity) and cytospin (Fig. 7E,F, respectively) revelated that PnV induced a significant increase in neutrophils compared to control. Analysis of NK cells (NK1.1 positive) showed that PnV increased the percentage cells, compared to control (Fig. 7F–H). MTX did not change neutrophils and NK cells from spleen in comparison to untreated group.

### Treatment with PnV increased the number of tumor associated macrophages (TAMs)

**Figure 8:**
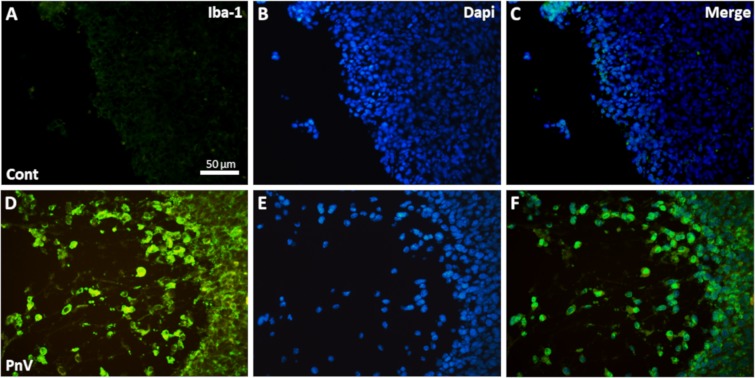
Immunostaining of the macrophage marker (Iba-1) in tumor sections. (**A–C**) Sections of a control (untreated) animal. (**D–F**) Mouse treated with PnV. Note that the control group did not show Iba-1-positive cells. On the other hand, sections of the PnV-treated animal have a large number of macrophages. All figures have the same scale (bar shown in panel A only).

Analysis of Iba-1 positive cells (a macrophage marker) within and around the tumor mass revealed that in the control tumor there were no macrophages (Fig. 8A–C). However, in the PnV treated mice, a large number of positive cells was present in the surrounding tissue, in the periphery of the tumor and within the tumor mass (Fig. 8D–F).

### *In vitro* macrophages were activated by PnV

**Figure 9:**
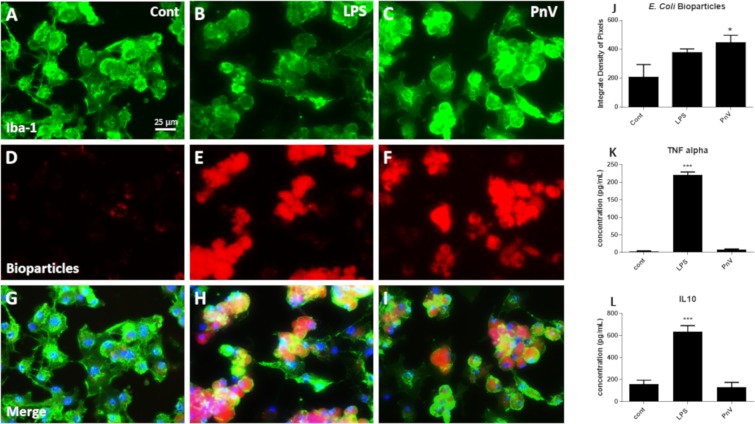
Phagocytosis assay using red E. coli pHrodo bioparticles; Immunofluorescence for Iba-1 (green) was used as a macrophage marker and the nuclei were stained with DAPI (blue). (**A**,**D** and **G**) Control (untreated cells) showed very little phagocytic activity; B - I: LPS and PnV treatments stimulated phagocytosis compared to control. Graph J shows the fluorescence quantification and statistical analysis. ELISA quantifications are demonstrated in graphs K and L. LPS induced increased amounts of TNF-alpha and IL-10, while PnV did not alter these cytokines compared to control. All figures have the same scale (bar shown in panel A only).

Fluorescent bioparticle phagocytosis assay (Fig. 9) showed that venom-treated macrophages (panels C, F and I) were more activated than untreated cells (panels A, D and G); LPS, used as a canonic positive control of macrophages activation to M1 profile, also induced increased phagocytosis (panels B, E and H), however cells treated with PnV were even more active (See graph J in Fig. 9). Figure 9 shows Iba-1 labeled cells (panels A–C), fluorescent bioparticles (panels D–F) and Iba-1/bioparticles/DAPI merged images (G–I). ELISA measures (graphs K and L in Fig. 9) demonstrated that while LPS induced increased TNF-alpha and IL-10 release, as expected, PnV did not change these cytokines.

### Several subfractions obtained from PnV have cytotoxic effect on GB cells

**Figure 10:**
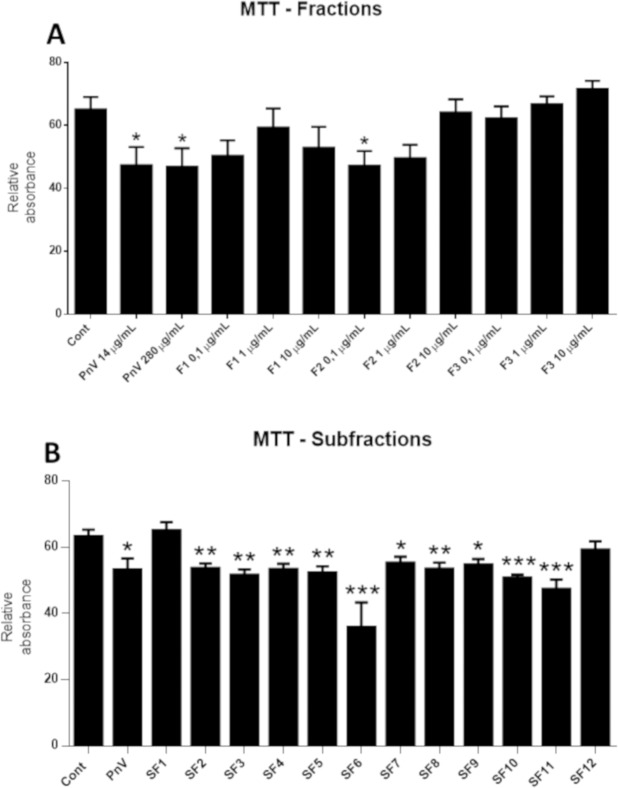
Cell viability assay (MTT). Graph A shows the effects of PnV (14 and 280 µg/mL) and three venom fractions (F1, F2 and F3: < 3, between 3 and 10 and> 10 KDa, respectively; 0.1, 1 and 10 µg/mL) on GB (NG97) cells *in vitro*. F1 and F2 at 0.1 µg/mL were more effective in decreasing cell viability, while F3 had no effect. Graph B shows subfractions (SF1-SF12; 0.1 µg/mL) isolated from F1 and F2. Except SF1 and SF12, all subfractions decreased cell viability, with SF6, SF10 and SF11 being the most significant.

The cell viability assay, showed in Fig. 10A, demonstrated that F1 and F2 (low and intermediate wight components) were cytotoxic to NG97 cells (F2 had statistical significance). F3 had no effects on GB cells. From the three tested concentrations, 0,1 µg/mL was the most significant. SF1-SF12, from F1 and F2 purification, were tested only at the concentration of 0,1 µg/mL (Fig. 10B) and showed that only SF1 and SF12 had no effected on the viability of cells, comparing to control. Nine subfraction, SF2-SF11, demonstrated anti-tumor effect, being the SF6, SF10 and SF11 the most effective comparing to control cells.

## Discussion

Recently, our group showed that *P. nigriventer* spider venom is cytotoxic to tumor cells *in vitro*, especially for NG97 GB cell line^7,14^. As follows, in the present work we aimed to investigate whether the systemic administration of the venom would alter the development of xenogeneic GB (NG97) implanted in mice. Females were used because the tumor development was more uniform than in males. The reason for this is unclear. The dose of PnV was chosen after preliminary tests, where other doses (850, 500, 200, 100 and 50 µg/Kg) were tested. Previous studies have shown that 850 µg/Kg was a sublethal dose in rats, however promoting several signs of intoxication (limb paralysis, convulsion, tremors, sialorrhea, sometimes death). For this study, the highest dose among the five tested, which did not cause any clinical signs of intoxication in any animal, was chosen (100 µg/Kg). In addition, methotrexate (MTX) is a chemotherapeutic drug used to treat various types of cancer^22^; Although not used for GB because the drug does not cross the BBB^23^, it is a classic chemotherapy that induces immunosuppression. Therefore, in this work, MTX was used as a control only to compare with the effects of PnV in relation to the effects on the immune cells.

The results demonstrated that PnV chemotherapy arrested tumor development. In addition, PnV tumors did not uptake detectable amounts of ^18^F-FDG, suggesting impairment of the metabolism of tumor cells. High glucose uptake is a hallmark of cancer; FDG uptake was thought to indicate the aggressiveness of tumors and SUV is a well-known measure for FDG uptake in PET/CT^24^. Therefore, the lower uptake of FDG may indicate that the tumors of PnV treated animals were less aggressive compared to control.

It is relevant to report that in this study only one animal treated with PnV, instead of developing a tumor in the back (site of inoculation of glioma cells), developed a tumor mass in the brain. This was a unique case and the cause is unclear. However, the possibility that this may have happened because PnV is able to permeabilize the BBB has to be considered for future therapeutic use of molecules obtained from the venom, since metastasis can be facilitated by the opening of the barrier. In addition, previous studies have shown that, although the venom promotes BBB breakdown in specific regions, changes in cell morphology or necrosis were absent in the central nervous tissue^25^. On the other hand, PnV stimulates neurons and glia^13,26^, which may be related to the seizures observed in envenomed rats and humans. An important observation is that, with the dose used in this study, the mice did not present convulsion or any signs of intoxication. Nevertheless, venom is a mixture of molecular entities, and that with antineoplastic effects are hopefully to be different from those with target in the BBB, neuron and glia; therefore, when the molecules have been isolated, these aspects have to be examined and the effects are expected to be more specific towards cancer cells.

Along with decreased tumor development, a better prognosis was observed in mice treated with PnV; the animals presented lower mortality in relation to the control and better recovery of weight. Nevertheless, PnV administration increased GOT levels in the serum, a marker of hepatic dysfunction. As same as before, we expect that purification of tumor active molecules may abolish hepatotoxic effect.

Histopathology showed that animals from control group had polymorphic hypercellular tumors, as expected. Other typical features were also present in this group, such as vascular proliferation, necrosis (with tumor cell palisading) and more than one cell type^27,28^. However, the majority of the tissue area in the PnV tumor was necrotic, lacking the histopathological characteristics typical of GB. This may explain the low uptake of ^18^F-FDG in PnV tumors.

To assess the systemic effects of venom, blood cells were analyzed, showing that PnV increased monocytes and did not change neutrophils. This effect is different from that observed in classical chemotherapies, which induce a decrease of immune cells. In the present study, MTX was used only to compare the effects of PnV with a classical immunosuppressor chemotherapeutic drug. MTX induced a neutropenia, as expected, while it did not affect monocytes. It is reported that MTX can affect hematopoiesis, causing leukopenia, anemia, and/or thrombocytopenia^29^. Corroborating with the literature, it was shown here that while PnV did not alter erythrocytes, hemoglobin and hematocrits levels, in animals treated with MTX all these parameters were reduced. On the other hand, while PnV did not change platelets count, MTX treatment increased. The literature often reports a decrease in platelets (thrombocytopenia)^22^ induced by MTX; however, thrombocytosis developed due to the use of MTX or MTX combined with other drugs has already been described^30–32^.

Considering the alterations induced by PnV in the blood immune cells, we decided to examine the profile of the immune cells isolated from the spleen. Interestingly, PnV did not change total macrophages, while decreased activated (IFN-γ positive) and suppressive (IL-10 positive) cells. Although PnV have decreased the percentage of activated and suppressors macrophages isolated from the spleen, many macrophages were detected in the tumor of animals treated with the venom, whereas they were not observed in the control. These results suggest that PnV induced an increase in blood monocytes, and these cells were directed preferentially to the tumor. Hypoxia is a common characteristic of many solid tumors and limited oxygen diffusion generates a gradient of oxygen availability from the blood vessel to the interstitial space and may underlie macrophages recruitment, promoting cancer progression^33^. The macrophages can be recruited to the tumor in PnV-treated animals because the tumor is highly necrotic and the environment is probably very poor of oxygen.

Macrophages in the tumor are known as Tumor Associated Macrophages (TAMs); TAMs exist in a spectrum, exhibiting spatiotemporal plasticity in regard to the phenotype^34,35^. In a simplified way, these cells can be divided into two subtypes, M1 and M2. Canonically, M1 is associated with pro-inflammatory properties and has antitumor effects, while M2 is associated with anti-inflammatory properties and promotes tumor growth and resistance to therapy^36^. Nevertheless, increasing evidence suggests that both contribute to the tumorigenesis and tumor cell migration/metastasis^37^. TNF-alpha and IL-12 are cytokines able to characterize M1, whereas IL-10 and IL-4 are able to characterize M2 phenotype^35,38^. Considering that PnV induced a decrease in polarized macrophages, both activated (INF-γ positive) and suppressed (IL-10 positive), it is possible that the profile of PnV-macrophages are phenotypically different of typical TAMs (M1 and M2) and may be more effective in fighting the tumor without promoting tumorigenesis.

It is important to mention that RAG^−/−^ mice do not have the complete immune response (lacking T lymphocytes) and the immunomodulatory effects of PnV on other systems need to be tested. Therefore, to confirm and better understand the effects of the venom, cultured macrophages generated from mice bone marrow were treated with PnV. The venom activated the cells, which became more phagocytic than control (untreated). LPS, used as a positive control of M1 induced profile^21^, also increased phagocytosis. On the other hand, while LPS enhanced TNF-α and IL-10 cytokines release, as expected^39^, PnV-phagocytic macrophages did not produce those pro- or anti-inflammatory cytokines, corroborating the *in vivo* findings. The PnV-activated macrophages profile seems to be different of M1 or M2 phenotype. The effects of PnV and its molecules in the immune system needs to be better investigated; experiments in immunocompetent mice and in a 3D spheroid coculture system are in course.

A previous report^40^ showed that P/Q- and N-type voltage-gated calcium channels (VGCC) inhibitors PhTx3-3 and Phα1β, from PnV, have inhibitory effects on proliferation and viability and evoked cell death of glioma cell lines (M059J, U138MG and U-251MG). Furthermore, Phα1β caused significant reduction of tumor areas *in vivo* and this toxin also increased GFAP-activated astrocytes and Iba-1 positive microglia in the peritumoral region, showing a modulation of these immune cells, which corroborates current findings. It has been shown here that purified fractions and subfractions of the PnV have antitumor action, decreasing the viability of GB cells; therefore, it is probable that not only calcium channel inhibitors are active in GBs. It is important to mention that the cytotoxic effect of PnV and most SFs, although significant, was not very pronounced. Our hypothesis is that this effect is added to the immunological activation against the tumor, resulting in a synergistic therapeutic action, justifying the strong tumor reduction observed in this study in the *in vivo* model. We are isolating and characterizing the immunomodulatory and antitumor PnV molecules. In addition, components of the venom with anti-migratory effects in GB cells are being identified.

In conclusion, the venom was effective in impairing tumor development in a murine xenogeneic model. It has also been demonstrated for the first time that the venom has immunomodulatory effects, and this may be an essential mechanism of the antineoplastic role of the venom components. The effects of PnV on immune cells suggest that its components did not induce immunosuppression, like classical chemotherapeutic drugs. In addition, PnV treatment increased macrophages into and surround the tumor, however with a different profile of M1. Studies in progress are selecting the venom molecules with antineoplastic and immunomodulatory effects and trying to better understand their mechanisms. The identification and synthesis of antineoplastic/immunomodulatory molecules can lead to a new multitarget chemotherapy.

## Supplementary information

Supplementary information.

## Data Availability

The authors declare that there is no restriction to make available the data and protocols used in this manuscript.

## References

[1] Thakkar JP (2014). Epidemiologic and molecular prognostic review of glioblastoma. Cancer Epidemiol. Biomarkers Prev..

[2] Ostrom QT (2013). CBTRUS statistical report: primary brain and central nervous system tumors diagnosed in the United States in 2006–2010. Neuro Oncol..

[3] Smoll NR, Schaller K, Gautschi OP (2013). Long-term survival of patients with glioblastoma multiforme (GBM). J. Clin. Neurosci..

[4] Alifieris C, Trafalis DT (2015). Glioblastoma multiforme: Pathogenesis and treatment. Pharmacol. Ther..

[5] Patridge E, Gareiss P, Kinch MS, Hoyer D (2016). An analysis of FDA-approved drugs: natural products and their derivatives. Drug Discov. Today..

[6] Escoubas P (2006). Molecular diversification in spider venoms: a web of combinatorial peptide libraries. Mol. Divers..

[7] Rapôso C (2017). Scorpion and spider venoms in cancer treatment: state of the art, challenges, and perspectives. J. Clin. Transl. Res..

[8] Lima, M. E. *et al* Phoneutria nigriventer Venom and Toxins: A Review. In: Gopalakrishnakone, P., Corzo, G. A., Lima, M. E. & Garc, E. D., Ed. Spider Venom. Netherlands: Springer, 1–24 (2015).

[9] Cruz-Höfling, M. A., Tavares, J. C. & Rapôso, C. Phoneutria nigriventer venom: action in the central nervous system. In: Gopalakrishnakone, P., Corzo, G. A., Lima, M. E. & Garc, E. D., Ed. Spider Venom. Netherlands: Springer, 175–202 (2015).

[10] Rapôso C, Zago GM, Silva G, Cruz-Höfling MA (2007). Acute blood brain barrier permeabilization in rats after systemic Phoneutria nigriventer venom. Brain Res..

[11] Rapôso C (2012). Effect of Phoneutria nigriventer venom on the expression of junctional protein and P-gp efflux pump function in the blood-brain barrier. Neurochem. Res..

[12] Rapôso C (2014). Triggering of protection mechanism against Phoneutria nigriventer spider venom in the brain. Plos One..

[13] Rapôso C (2016). Neuropharmacological effects of Phoneutria nigriventer venom on astrocytes. Neurochem. Int..

[14] Santos NB (2018). Venom of the Phoneutria nigriventer spider alters the cell cycle, viability and migration of cancer cells. J. Cell Physiol..

[15] Grippo MC, Penteado PF, Carelli EF, Cruz-Höfling MA, Verinaud L (2001). Establishment and partial characterization of a continuous human malignant glioma cell line: NG97. Cell Mol. Neurobiol..

[16] Schenka AA (2005). Immunophenotypic and ultrastructural validation of a new human glioblastoma cell line. Cell Mol. Neurobiol..

[17] Machado CM (2005). Morphological characterization of a human glioma cell line. Cancer Cell Int..

[18] Machado CM (2008). Characterization of cells recovered from the xenotransplanted NG97 human-derived glioma cell line subcultured in a long-term *in vitro*. BMC Cancer..

[19] Machado CM (2009). Ultrastructural characterization of the new NG97ht human-derived glioma cell line using two different electron microscopy technical procedures. Micrcosc. Res. Tech..

[20] Marim FM, Silveira TN, Lima DS, Zamboni DS (2010). A method for generation of bone marrow-derived macrophages from cryopreserved mouse bone marrow cells. PLoS One..

[21] Vogel DY (2014). Macrophages migrate in an activation-dependent manner to chemokines involved in neuroinflammation. J. Neuroinflammation..

[22] Campbell JM (2016). Methotrexate induced toxicity pharmacogenetics: an umbrella review of systematic reviewers and meta-analyses. Cancer Chemother. Pharmacol..

[23] Cooper I (2015). Combined local blood-brain barrier opening and systemic methotrexate for the treatment of brain tumors. J. Cereb. Blood Flow Metab..

[24] Shangguan C (2018). Cancer-associated fibroblasts enhance tumor 18F-FDG uptake and contribute to the intratumor heterogeneity of PET-CT. Theranostics..

[25] Le Sueur LP, Kalapothakis E, Cruz-Höfling MA (2003). Breakdown of the blood-brain barrier and neuropathological changes induced by Phoneutria nigriventer spider venom. Acta Neuropathol..

[26] Cruz-Höfling MA, Zago GM, Melo LL, Rapôso C (2007). c-FOS and n-NOS reactive neurons in response to circulating Phoneutria nigriventer spider venom. Brain Res. Bull..

[27] Pathpedia – e-Atlas Histopathology/Brain & cord. Glioblastoma (GBM). ID: BR006.1. PathPedia, LLC © 2006-2019, https://www.pathpedia.com/education/eatlas/histopathology/brain_and_cord/glioblastoma_(gbm).aspx (2019).

[28] Habberstad AH, Lind-Landström T, Sundstrøm S, Torp SH (2012). Primary human glioblastomas - prognostic value of clinical and histopathological parameters. Clin. Neuropathol..

[29] Wang W, Zhou H, Liu L (2018). Side effects of methotrexate therapy for rheumatoid arthitis: A systematic review. Eur. J. Med. Chem..

[30] Kaydok E, Salbas E (2018). Thrombocytosis development due to the methotrexate in a patient with rheumatoid arthritis. Rheumatology (Sunnyvale)..

[31] Ravelli A (1999). The extended oligoarticular subtype is the best predictor of methotrexate efficacy in juvenile idiopathic arthritis. J. Pediatr..

[32] Kenis Y, Michel J, Tagnon HJ (1970). Thrombocytosis, methotrexate, and citrovorum factor. The Lancet..

[33] Campillo N (2019). Differential oxygenation in tumor microenvironment modulates macrophages and cancer cell crosstalk: novel experimental setting and proof of concept. Front. Oncol..

[34] Ginhoux F, Schultze JL, Murray PJ, Ochando J, Biswas SK (2016). New insights into the multidimensional concept of macrophage ontogeny, activation and function. Nat. Immunol..

[35] Mosser DM, Edwards JP (2008). Exploring the full spectrum of macrophage activation. Nat Rev. Immunol..

[36] Rodell CB (2018). TLR7/8-agonist-loaded nanoparticles promote the polarization of tumour-associated macrophages to enhance cancer immunotherapy. Nat. Biomed. Eng..

[37] Xiao M, Zhang J, Chen W, Chen W (2018). M1-like tumor-associated macrophages activated by exosome-transferred THBS1 promote malignant migration in oral squamous cell carcinoma. J. Exp. Clin. Cancer Res..

[38] Gordon S, Martinez FO (2010). Alternative activation of macrophages: mechanism and functions. Immunity..

[39] Kelly B, Tannahill GM, Murphy MP, O’Neill LA (2015). Metformin inhibits the production of reactive oxygen species from NADH:ubiquinone oxidoreductase to limit induction of interleukin-1β (IL-1β) and boosts interleukin-10 (IL-10) in lipopolysaccharide (LPS)-activated macrophages. J. Biol. Chem..

[40] Nicoletti NF (2017). Pre-clinical evaluation of voltage-gated calcium channel blockers derived from the spider P. nigriventer in glioma progression. Toxicon..

