# Supplementary material for: Spider venom administration impairs glioblastoma growth and modulates immune response in a non-clinical model
Source: Sci Rep. 2020 Apr 3;10:5876. doi: 10.1038/s41598-020-62620-9 (PMC7125223; doi:10.1038/s41598-020-62620-9)
Supplement: Supplementary file 1 — Supplementary information. [file 41598_2020_62620_MOESM1_ESM.docx]

**Spider venom administration impairs glioblastoma growth and modulates immune response in a non-clinical model**

Amanda Pires Bonfanti^1,2ϯ^, Natália Barreto^1,2 ϯ^, Jaqueline Munhoz^1,2^, Marcus Caballero^1,2^, Gabriel Cordeiro^1,2^, Thomaz Rocha‐e‐Silva^3^, Rafael Sutti^4^, Fernanda Moura^5^, Sérgio Brunetto^6^, Celso Dario Ramos^7^, Rodolfo Thomé^8^, Liana Verinaud^2^, Catarina Rapôso^1*^

**SUPPLEMENTARY INFORMATION**

**
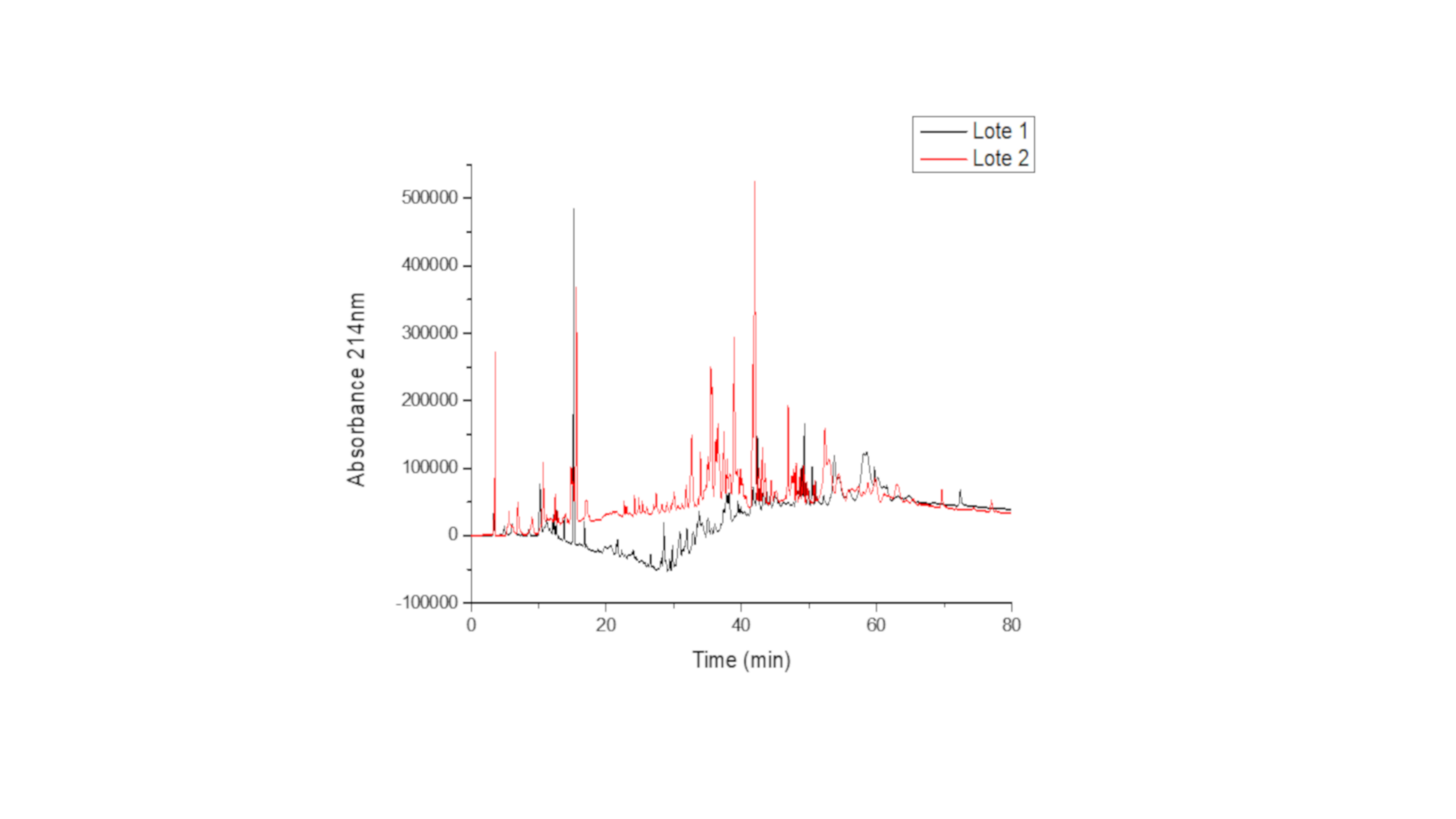
**

**Supplementary Figure 1**. The PnV profile obtained by high – pressure liquid chromatography (HPLC) showed that there were no relevant differences between the two pooled venom samples used in this work.

**
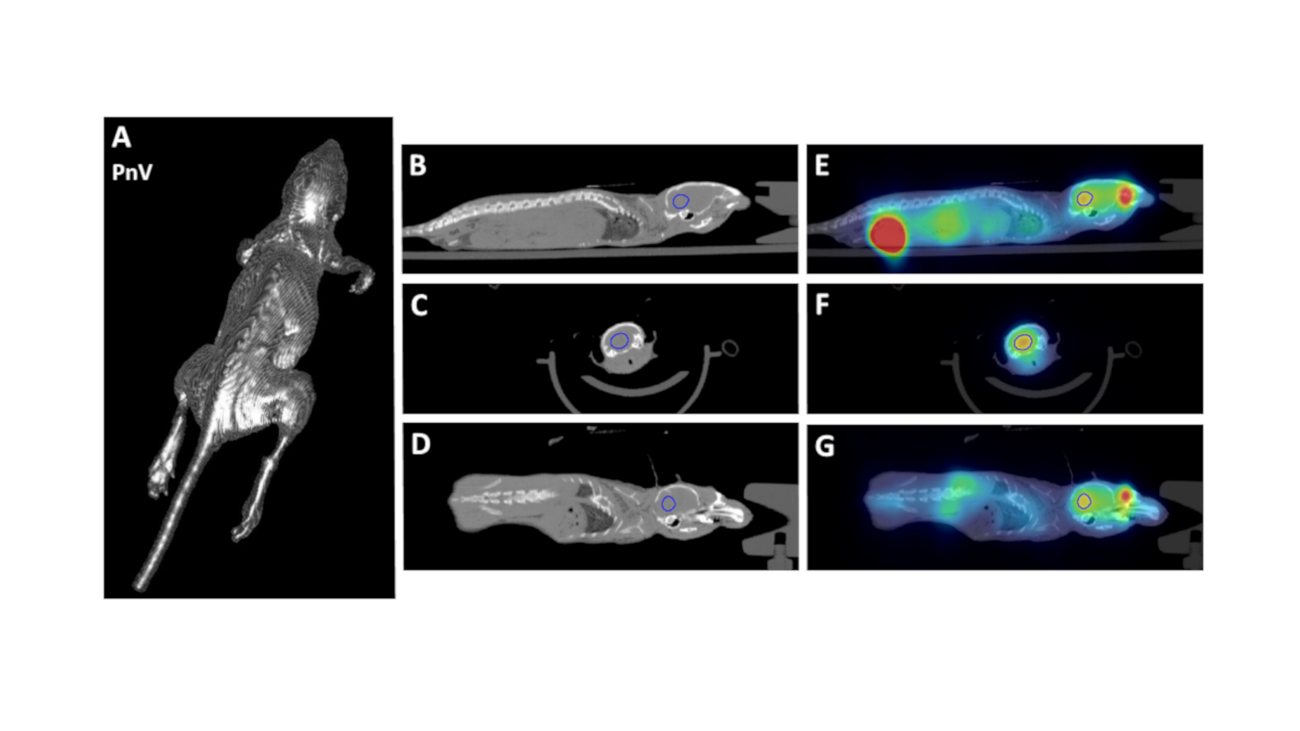
**

**Supplementary Figure 2**. Positron Emission Tomography-Computed Tomography (PET-CT) images of a RAG^-/-^ mouse treated with PnV. This animal did not develop tumor mass in the back (site of GB NG97 cells inoculation); however, it developed a brain tumor (circled area in panels B - G). This was a unique case. A - 3D reconstruction; B and E - Sagittal sections; C and F - Transversal sections; D and G - Coronal sections.

**Videos 1 and 2 represent a three-dimensional reconstruction of control (untreated) and PnV-treated animals, respectively, at the end of the experiment (21 days).**
